# Multi-Beam Scanning Electron Microscopy for High-Throughput Imaging in Connectomics Research

**DOI:** 10.3389/fnana.2018.00112

**Published:** 2018-12-11

**Authors:** Anna Lena Eberle, Dirk Zeidler

**Affiliations:** Carl Zeiss Microscopy GmbH, Oberkochen, Germany

**Keywords:** 3D volume EM, scanning electron microscopy, high-throughput imaging, high-content imaging, multibeam

## Abstract

Major progress has been achieved in recent years in three-dimensional microscopy techniques. This applies to the life sciences in general, but specifically the neuroscientific field has been a main driver for developments regarding volume imaging. In particular, scanning electron microscopy offers new insights into the organization of cells and tissues by volume imaging methods, such as serial section array tomography, serial block-face imaging or focused ion beam tomography. However, most of these techniques are restricted to relatively small tissue volumes due to the limited acquisition throughput of most standard imaging techniques. Recently, a novel multi-beam scanning electron microscope technology optimized to the imaging of large sample areas has been developed. Complemented by the commercialization of automated sample preparation robots, the mapping of larger, cubic millimeter range tissue volumes at high-resolution is now within reach. This Mini Review will provide a brief overview of the various approaches to electron microscopic volume imaging, with an emphasis on serial section array tomography and multi-beam scanning electron microscopic imaging.

## Introduction—The Decade of Brain Imaging

Just as “genomics” deciphers complete genomes of live beings since two decades ago, a new field aiming at fully deciphering the circuitry of the nervous system on a large scale is emerging now. This field has accordingly been coined “connectomics” (Sporns et al., 2005), and has similarly been able to attract growing research interest over the past years (BRAIN, 2013).

First descriptions of neuronal morphology and the idea that individual neurons are anatomically connected is already a century old (Cajal, 1899). In more recent times, information about the intrinsic connectivity of the nervous system has been obtained also by *in vivo* approaches such as electrophysiology (Mandonnet et al., 2010), diffusion tractography [DTI (Mori and Zhang, 2006; Hagmann et al., 2007; Guye et al., 2008)], functional magnetic resonance imaging [fMRI (Mamedov et al., 2012; Lowe et al., 2016)] or optical imaging (Petroll et al., 1994; Kleinfeld et al., 2011; Chen et al., 2015). None of these methods, however, reveals information about the neuronal connections at their ultrastructural level, which is expected to reveal deeper insights into how a nervous system functions (Morgan and Lichtman, 2013). The ambitious approach of connectomics is to gain an understanding of the circuitry of the brain by mapping every single component and trace every connection of a certain volume of brain tissue (Lichtman and Denk, 2011).

The actual dimension of the volume of interest depends on a number of factors, for example on the question to be answered, the model organism or the neuronal system to be investigated. Originally describing the connections of a complete nervous system, different methodologies with different spatial resolutions lead to the differentiation of the connectome into different scales: the microscale, the mesoscale, and the macroscale connectome. The macroscale connectome parcellates the brain with millimeter resolution into anatomically or functionally distinct brain regions (Fellemann and Van Essen, 1991) and usually is assessed by non-invasive measures, such as DTI (Beckmann et al., 2009) or fMRI (Nelson et al., 2010). On the mesoscale, neuronal populations with distinct anatomical (Mountcastle, 1997) or functional (Callaway and Katz, 1990) features are described at a spatial resolution of hundreds of micrometers (Zhao et al., 2005). Mapping the finest details on the cellular level corresponds to the microscale connectome (Bargmann and Marder, 2013, Schröter et al., 2017). Bridging the gaps between these different scaling levels might enable a more unified, multiscale description of the connectome.

With specific labeling (Young and Feng, 2004; Lakadamyali et al., 2012), light microscopical methods characterize individual neurons very well, while still offering relatively large sample volumes. The surrounding, unlabeled context, however, is usually missing. Even though the resolution limit of light microscopes has been improved over decades to well below the light wavelength (Klar et al., 2000; Betzig et al., 2006; Hell, 2007), the nervous system contains structures that are not easily resolvable with them. Hence, electron microscopy (EM) has become a commonly used technique to resolve ultrastructural details on a cellular scale.

If acquisition of a whole volume is required, the volume of interest needs to be sectioned physically (Ware, 1975) or optically (Minsky, 1988; Denk et al., 1990; Neil et al., 1997; Huisken et al., 2004; Santi, 2011) before it can be imaged in 2D. The 2D data sets are stacked and aligned in the third dimension, and all individual compartments are usually labeled on the 2D images first and then tracked throughout the volume. This method in the end yields a “dense reconstruction” (Kasthuri et al., 2015) and will answer important questions about the general principles how neurons connect: Does a minimal repetitive circuitry unit or motif exist? How do different brain regions compare, and how does this relate to differences between individuals and species (Womelsdorf et al., 2014, Borst and Helmstaedter, 2015, Lee et al., 2016)? Based on such data for healthy brain tissue, the next step is to learn about deviations in pathological conditions. Are there structural changes in brains with neurodegenerative diseases, and how does this knowledge help to develop novel treatments? However, before such information can be derived, imaging of the volume of interest needs to be accomplished.

## Overview of Different Approaches to Volume Electron Microscopy

Over the last decades, several methods for volume electron microscopy have been established (Briggman and Bock, 2012; Kremer et al., 2015; Mikula and Denk, 2015; Titze and Genoud, 2016). The nature of the experiment determines which method is optimally suited.

One main differentiator is which part of the tissue block is imaged—the cut-off and collected ultra-thin section or the freshly exposed block surface after a cut. The main advantage of collecting serial sections is that the sample is preserved and can be imaged repeatedly. Reconstruction of the volume after imaging is challenging, as the data needs to be corrected for distortion and translation occurring during the cutting process (Saalfeld et al., 2010). The section thickness is limited down to ~30 nm, leading to non-isotropic voxels when images are acquired with a smaller lateral pixel size. With block-face imaging, the reconstruction of the final data set needs less distortion and translation corrections, because the acquired area is always the same in shape and position. As the sample is lost in the sectioning process, advanced control of the imaging step has to ensure each section is acquired with sufficient quality before moving on to the next cutting step (Binding et al., 2013).

Classically, serial ultrathin sections have been prepared using an ultramicrotome, followed by manually placing them onto copper grids for imaging in a transmission electron microscope [TEM (Harris et al., 2006)]. Recent developments regarding automation on the sample handling as well as the imaging part enabled relatively large-scale sample volumes to be imaged and reconstructed (Zheng et al., 2018). However, the standard TEM grid ultimately limits the size of the accessible volume to a maximum of 1 × 2 mm (Briggman and Bock, 2012). For samples exceeding this size, placement of the sections on a solid substrate is necessary, which in turn requires imaging in a scanning electron microscope (SEM). In principle, classical sample preparation schemes suitable for SEM imaging can be used (Echlin, 2009), however, such protocols might need to get slightly adapted to accommodate larger tissue volumes (Hua et al., 2015; Mikula and Denk, 2015).

For preparation of large series of consecutive sections, the automated ultramicrotome [ATUMtome (Hayworth et al., 2006; Schalek et al., 2011)] uses a conveyer belt type mechanism to automatically pick up sections on a tape right after they have been cut. ATUMtome has been reported to reliably collect thousands of consecutive sections (Hayworth et al., 2014), enabling sectioning of large sample volumes. Several tape materials with different physical properties have been evaluated so far (Kubota et al., 2018). For manual preparation of a small to medium number of sections, a micromanipulator setup with an advanced substrate holder [ASH (Spomer et al., 2015)] is sufficient. This is especially useful with small samples and for the preparation of ribbons of sections (Wacker et al., 2015). Sections can be placed directly onto a silicon wafer as substrate, which is advantageous for imaging in a SEM, or onto indium tin oxide coated coverslips, such that imaging with light microscopes is possible as well. Further developments aim at more efficient handling and placement of serial sections. For example, it has been demonstrated that sections can be controlled magnetically while still floating in the water bath after cutting (Templier, 2017). By tracing each individual slice to its position on the final substrate, this technique allows dense packing of sections onto the wafer, increasing the degree of automation by reducing on the total number of sample carriers.

For block-face imaging, the *in-situ* microtome (Denk and Horstmann, 2004) and focused ion beam ablation (Knott et al., 2008; Xu et al., 2017) are the two most established methods. The *in-situ* microtome allows for rather quick ablation of the surface of medium sized sample volumes of up to (0.5 × 0.5 × 0.5) mm^3^. It has been reported to run autonomously for up to several weeks (Wanner et al., 2015). Yet, the section thickness is limited by the radius of the knife edge. About 20 nm are achievable because the sections do not need to be collected, but the thinner a section thickness is chosen, the less reproducible the results will be. If isotropic voxel data with <15 nm side length is required, FIB-SEM is currently the only available technique. The slow ablation speed results in only rather small accessible volumes, currently few tens of micrometers side length at maximum. A recent development aims to increase the throughput of this approach by dissecting the sample with a hot knife without loss into smaller cubicles and parallelizing the ablation/imaging process with several FIB-SEM systems (Hayworth et al., 2015). The acquired datasets are subsequently recombined into the original sample volume. Even tracing of fine neuronal processes seems to be possible across the borders of these cubicles.

## Sample Volumes and Imaging Time

Comparison tables providing an overview of the various electron microscopical approaches to volume imaging are available in the review articles from Briggman and Bock (2012), Titze and Genoud (2016), and Kornfeld and Denk (2018). However, the limiting factor for all approaches is the sample volume that can be assessed in a reasonable time and at the required resolution. Thus, all volume electron microscopy applications will benefit from an increase in image acquisition throughput. As neuronal circuits can span hundreds of micrometers or more, the need for imaging larger sample volumes is particularly important within the field of connectomics.

For example, some types of neurons, such as Claustrum neurons, reportedly can wrap around the whole brain (Reardon, 2017). In the case of an entire circuit, the cortical column (Mountcastle, 1957; Fox, 2018) describes the concept of a modular building block of circuitry. It spans all six cortical layers and part of the underlying white matter, therefore measuring to a depth of up to 2 mm and a lateral extent of up to 500 μm. Such volumes can easily exceed the capabilities of a standard single beam scanning electron microscope—or rather the time that can be allocated for such a project. One cubic millimeter of brain tissue, cut into 30 nm thin slices, results in more than 33,000 sections of 1 mm^2^ each. Imaging this total area with a pixel size of 4 nm, which is sufficient to grasp all required details, will take approximately 12 years with a state-of-the-art single-beam SEM (Titze and Genoud, 2016).

However, accelerating the imaging with a single-beam SEM will have an impact on the image quality (Reimer, 1998): Increasing the scan speed of the illuminating electron beam will result in reduced contrast to noise ratio due to the shorter dwell time per pixel, i.e., less electrons per pixel. This can be compensated by increasing the beam current at the cost of decreasing the resolution of the illuminating electron beam due to electron-electron interactions. The solution to this dilemma is parallelizing the imaging process. In principle, one could use several SEMs in parallel; a more economical way is to parallelize imaging within a single instrument. Multi-beam scanning electron microscopes (Ren et al., 2014; Eberle et al., 2015a) will enable data acquisition times of less than half a year in the example above and might therefore help bridging the gap between microscale- and mesoscale-connectome.

## Multi-beam Scanning Electron Microscopy

Using multiple electron beams in parallel has been of interest in electron beam lithography for decades: reducing the writing time of semiconductor structures with multiple electron beam lithography is of great economic interest (Pease, 1979; Chang et al., 2001; Platzgummer et al., 2013). If, next to multiple-beam illumination, multi-beam imaging is also required, a detection path needs to be added. Up to date, there are a number of different concepts for multi-beam electron microscopes, such as multi-column or multi-beam systems (Mukhtar, 2018). The multi-column approach proposes multiple miniaturized electron optical columns in parallel (e.g., Meisburger et al., 2015). The number of micro-columns that have been proposed is for example 69 in Luo and Khursheed (2014). The multi-beam approach utilizes a bundle of electron beams generated from a single electron source and a single column (Mohammadi-Gheidari and Kruit, 2011; Keller et al., 2014).

What speed advance does a multi-beam SEM provide? Theoretically, the imaging throughput of a multi-beam SEM equals that of a comparable single-beam SEM multiplied by the number of beams. For the single beam SEM, the area throughput is basically given by pixel dwell time and total number of pixels to be acquired. Overhead times, such as stage movements, are usually of minor impact, especially for single-beam SEMs with large frame stores that allow to tesselate an area with fewer individual, but very large image tiles that take quite long to acquire. For multi-beam SEMs, the pure imaging time for a similarly large image tile consisting of many sub-images is reduced by the above mentioned theoretical factor. If the overhead remains unaltered, its relative impact on total acquisition time will increase.

**Figure 1:**
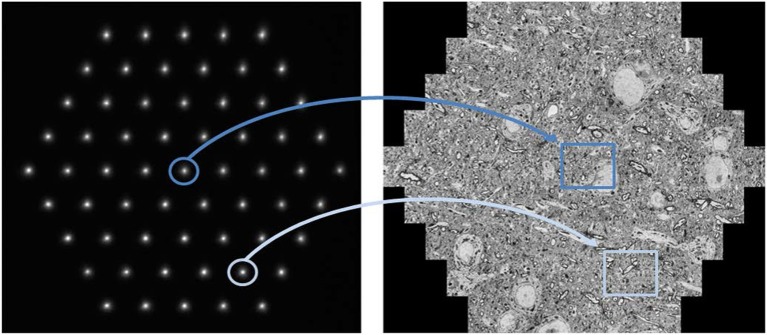
Multi-beam SEM principle of operation: The left image shows the signal electrons in the form of 61 secondary electron spots at the detector plane. Each spot corresponds to one secondary beam that is collected in an individual channel and acquired by one detector. All illuminating electron beams are scanned concurrently which leads to fluctuations in the signal intensity of the signal electron spots shown here. These changes are detected and related to the location of the sample the signal stems from. As a result, the beams marked in dark and light blue simultaneously acquire the images marked in dark and light blue, respectively. The right image shows a montage of the 61 single beam images recorded in one shot with a total field of view of about 110 μm. Sample with courtesy from Jeff Lichtman and Richard Schalek, Harvard University; figure adapted from Eberle et al. (2015b).

Under experimental conditions, an image acquisition rate of up to one terapixel per hour (Haehn et al., 2017) is achievable with a 61-beam SEM (Figure 1). While the illuminating beam array scans over the sample surface, secondary electrons are generated at each position of the primary electron beams. These are collected into separate channels, and the signal intensity is detected as function of the sample position of the primary electron beams. Pixel by pixel, the image for each individual beam is generated, and these images are merged to form the hexagonal full multi-beam field of view (mFOV, figure 1, right). If the region of interest (ROI) to be imaged is larger than one mFOV, the stage is moved to an adjacent sample position and the next mFOV is acquired with a little overlapping seam. The image information present in the overlap areas is used to correctly stitch together all mFOVs of an ROI. More details of the operating principle have already been described elsewhere (Eberle et al., 2015b; Kemen et al., 2015).

**Figure 2:**
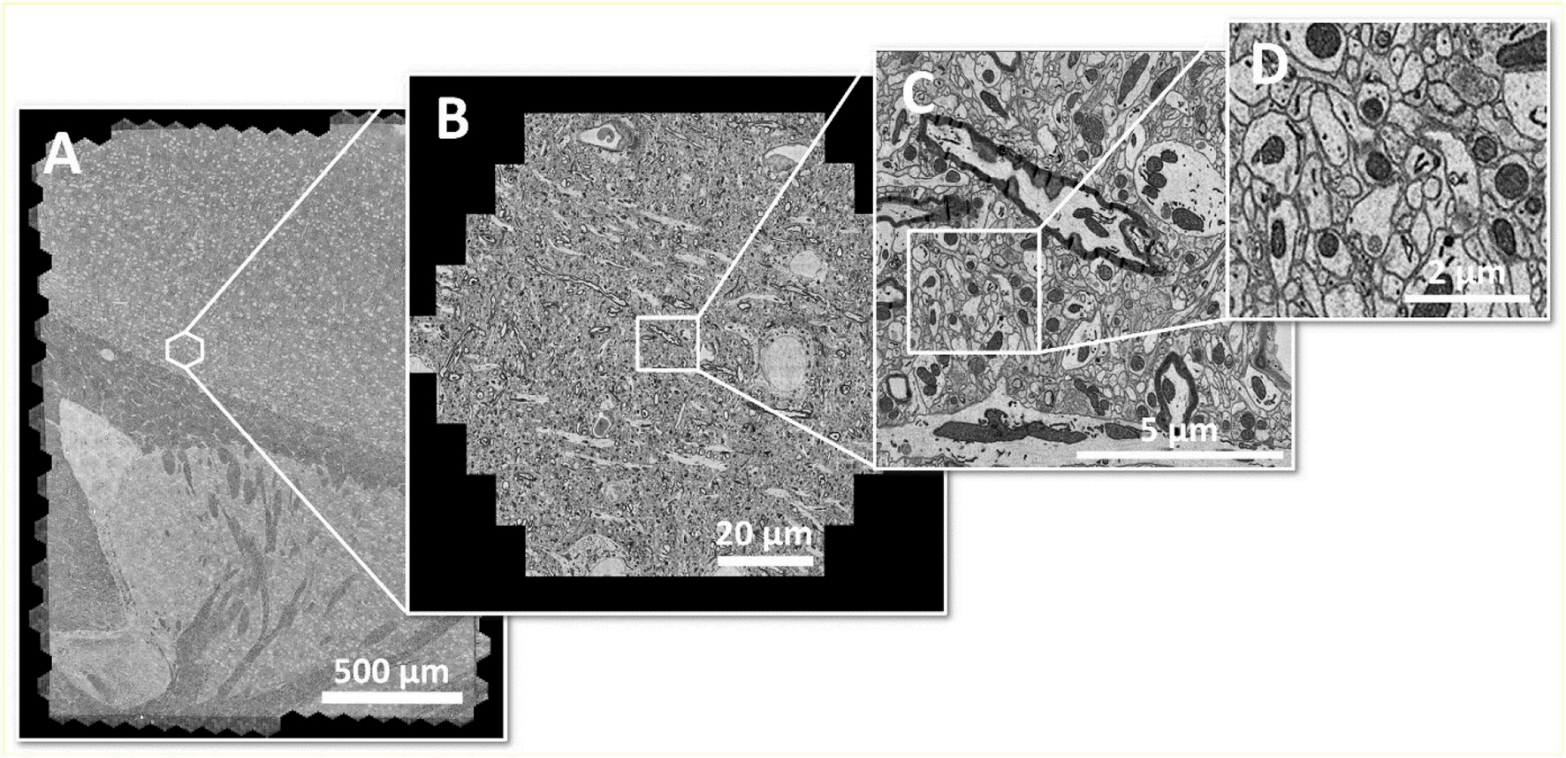
Large area imaging example: **(A)** Coronal mouse brain section from one hemisphere, fully acquired within 20 min with a 61-beam SEM at 4 nm pixel size, resulting in 290 GB of image data. The area of ~3 mm^2^ consists of 492 individual hexagonal fields of view **(B)** or 30,012 single-beam image tiles **(C)** in total. **(D)** shows an enlarged cutout. Sample with courtesy from Jeff Lichtman and Richard Schalek, Harvard University; figure adapted from Eberle et al. (2017).

It shall be noted, though, that the highest image acquisition speed is only useful if continuous operation can be guaranteed. In case of a multi-beam SEM, this requires high automation effort, as all beams of the multi-beam array need to perform equally in order to generate homogeneous image data across the full mFOV. Figure 2 shows an example of a seamlessly imaged mouse brain section of ~3 mm^2^ at 4 nm pixel size.

## Large Data Challenges

The overwhelming data rates high throughput EMs produce call for adequate strategies to handle this amount of data.

The simplest approach is to store the data on a local storage system as they are produced. This has the drawbacks of any local storage system, such as limited extendibility and data accessibility, limited simultaneous read/write operations, and backup effort. For small imaging volumes, nevertheless, the simplicity of this solution can still outweigh the drawbacks. Alternatively, the data can be stored in a distributed or even public storage system, with better scalability and accessibility. However, in that case, data transfer may become a significant cost factor.

Storage needs can be reduced when data compression may be applied. There is a tradeoff between data acquisition rate and data quality when imaging at highest data rates. Here, the task isb not to produce the best image with good contrast-to-noise ratio (CNR), but an image that can still be processed reliably. Depending on the application, highest data acquisition rates may be achieved at a point where images have a CNR inadequate for lossless data compression. If the application allows data compression with loss of information, larger compression factors are achievable. Next to general image compression methods, this might also be vectorization of data, e.g., by contour finding. With a typical single beam image size of 5–12 Mbyte, and a typical size of a vectorized data set of few kbytes to several 10 kbytes, the achievable data reduction rate is then about 10^2^-10^3^. In the case of contour finding, for example, this value depends on the number of features in the image and the required contour accuracy. The more a priori knowledge about the images is available, the better the image data can be condensed into a corresponding model.

Ultimately, real time data processing will allow for maximum data rate reduction. In the extreme case, each image could be reduced to e.g., one bit of information, depending on whether it matches a predefined criterion or not, and just storing this information. In general, a number of key parameters, corresponding to a few bytes, will be extracted from each image, and only these parameters need to be stored. The data reduction rate will then be on the order of 10^6^.

This extreme case is often not possible. Even worse, data amounts may increase during processing before they can be reduced. In a connectomics data set, for example, potentially billions of neurons need to be represented unambiguously, so 64 bit encoding is required initially. As the image data usually is encoded in 8 bit format, the segmentation data size exceeds the actual image size by a factor of 8. Only after segmentation, data will be compressed by a factor of about 700 (Haehn et al., 2017). This example shows that connectomics research today is forced to also focus onto the development of suitable processing solutions for such huge data sets.

## Outlook

The seamless integration of storage and computing solutions to the imaging system will be one of the future requirements for high throughput EM experiments in connectomics. Once this has been accomplished, further improving the imaging throughput of single-beam and multi-beam SEMs will enable investigating even larger volumes of neuronal circuitry. This becomes even more relevant if new sample preparation technologies with faster ablation and higher resolution, such as high-speed ion milling, become available (Nowakowski et al., 2017; Kornfeld and Denk, 2018).

For example, an ultrahigh-precision stage could reduce downstream computational efforts for seamless image stitching between adjacent mFOVs. Improving stage move times will reduce the imaging overhead. With improved contrasting of the sample, images could be taken at less electrons per pixel. This means, at constant or improved current per beam, faster scans would be possible. For several types of connectomics investigations, there will be a need for improved resolution. Multi-beam SEM technology has just recently become available and has the potential to fulfill the throughput and resolution requirements of future connectomics experiments needs.

Although both improved throughput, causing higher data rates, as well as better resolution, enabling smaller and therefore more voxels per volume, will pose an even larger challenge on the already limiting computational effort, the development of computation technology is expected to match the demands of connectomics research in the years to come. Manual tracing and segmentation (White et al., 1986) has been replaced by machine learning and neural networks trained with ground truth from manual segmentation (Turaga et al., 2010; Arganda-Carreras et al., 2015; Berning et al., 2015; Januszewski et al., 2016). The main task for the near future will be to implement the existing tools into scalable architectures.

## Author Contributions

All authors listed have made a substantial, direct and intellectual contribution to the work, and approved it for publication.

### Conflict of Interest Statement

Both authors are employed by the company Carl Zeiss Microscopy GmbH, Oberkochen.
